# A Bidirectional Brain-Machine Interface Algorithm That Approximates Arbitrary Force-Fields

**DOI:** 10.1371/journal.pone.0091677

**Published:** 2014-03-13

**Authors:** Alessandro Vato, Francois D. Szymanski, Marianna Semprini, Ferdinando A. Mussa-Ivaldi, Stefano Panzeri

**Affiliations:** 1 Department of Robotics, Brain and Cognitive Sciences, Istituto Italiano di Tecnologia, Genova, Italy; 2 Department of Physiology, Northwestern University, Chicago, Illinois, United States of America; 3 Department of Biomedical Engineering, Northwestern University, Evanston, Illinois, United States of America; 4 Sensory Motor Performance Program, Rehabilitation Institute of Chicago, Chicago, Illinois, United States of America; 5 Center for Neuroscience and Cognitive Systems @UniTn, Istituto Italiano di Tecnologia, Rovereto, Italy; 6 Institute of Neuroscience and Psychology, University of Glasgow, Glasgow, United Kingdom; University of Chicago, United States of America

## Abstract

We examine bidirectional brain-machine interfaces that control external devices in a closed loop by decoding motor cortical activity to command the device and by encoding the state of the device by delivering electrical stimuli to sensory areas. Although it is possible to design this artificial sensory-motor interaction while maintaining two independent channels of communication, here we propose a rule that closes the loop between flows of sensory and motor information in a way that approximates a desired dynamical policy expressed as a field of forces acting upon the controlled external device. We previously developed a first implementation of this approach based on linear decoding of neural activity recorded from the motor cortex into a set of forces (a force field) applied to a point mass, and on encoding of position of the point mass into patterns of electrical stimuli delivered to somatosensory areas. However, this previous algorithm had the limitation that it only worked in situations when the position-to-force map to be implemented is invertible. Here we overcome this limitation by developing a new non-linear form of the bidirectional interface that can approximate a virtually unlimited family of continuous fields. The new algorithm bases both the encoding of position information and the decoding of motor cortical activity on an explicit map between spike trains and the state space of the device computed with Multi-Dimensional-Scaling. We present a detailed computational analysis of the performance of the interface and a validation of its robustness by using synthetic neural responses in a simulated sensory-motor loop.

## Introduction

While the idea of connecting brains to machines has surfaced time and again [1], [2], the concept of a brain-machine interface (BMI) has developed as a mainstream research topic only more recently, building on progress in understanding how movement plans are encoded in motor cortical signals. Two main approaches have emerged. One approach is based on decoding motor cortical signals as a proxy for the intended state of motion [3], [4] or for muscle activations [5]. The other view [6] is based on decoding high-level motor goals from neural activity in areas such as the posterior parietal cortex, and to communicate this goal to an external artificial controller in charge of its execution. In both approaches the focus of the BMI is on decoding neural signals. It is only more recently that attention has been devoted to the dual problem of encoding in the brain information about the state of motion of external devices by using electrical stimulation [7]–[9].

This progress naturally calls for closing the loop between encoding and decoding, by combining in the same system a decoding interface that maps neural activities into commands to the external device and an encoding interface that maps the state of the device into neural signals using electrical stimulation [10]. Such closed-loop systems are potentially important both for clinical applications and as neuroscientific research tools for investigating neural plasticity by coupling a pattern of stimuli with the evoked responses through an external system with known dynamical properties.

Here, we present the findings of a computational investigation of a novel brain-machine interface architecture that proposes an explicit set of rules to coordinate the decoding and the encoding components of the interface. This set of rules implements control policies based on the closed-loop interaction between the motor commands expressed by neural activity and their sensory consequences, which are fed to the brain as encoded information about changes of state of the controlled device. These control policies are inspired by the physiological interaction between descending cortical signals and the activity of central pattern generators, where the descending commands modulate the timing and shape of the trajectories that emerge from the interaction between the limbs, the neural control system (including its voluntary components) and the environment [11]–[17].

A feedback control policy is a function that seeks to attain a goal while reacting to unexpected circumstances. Sutton and Barto [18] define such a policy in more formal terms as “a mapping from perceived states of the environment to actions to be taken when in those states” given a predefined goal. Translated into mechanical terms and in the framework of movement control, a motor-control policy can be represented as a force field: a force (the action) that, for a given goal, the control system generates as a function of the observed state of motion of the controlled object. Force fields have the inherent property that any field shape can be produced or approximated by summation of other force fields. This is an important prerequisite for modularity, as it provides a simple mechanism to build a repertoire by combining a set of primitive elements [19], [20] [19], [20]. A key requirement to that effect is, for the primitive policies, to be mathematically equivalent to basis functions with structure rich enough to approximate other policies of arbitrary form. Another important aspect of force fields is that the concept of “behavior” is not reduced to a particular trajectory of the controlled element, but includes a whole family of trajectories. The interaction between the field and the controlled object generates a family of trajectories, one for each point of the state space. The control system may select a particular trajectory by setting an initial state and letting the dynamical interactions between controlled system and environment shape the temporal evolution of movement.

In recent work, we took inspiration from this view of biological motor control to first conceptually propose [10] and then implement in anesthetized rats [21] a bidirectional BMI capable of generating a force field with a given desired structure acting upon the controlled mechanical system. In the first implementation of this approach, we developed a method based on linear decoding of recorded motor cortical activities into force vectors applied to a point mass and encoding the position of the point mass into patterns of electrical stimuli delivered to the somatosensory cortex. A major limitation of this previous algorithm was that it could only approximate invertible force fields - that is invertible position-to-force maps.

Here we overcome this limitation by introducing a new form of bidirectional interface, which we call the *non-linear dynamic brain-machine interface (ndBMI*) that approximates a virtually unlimited family of fields. The interface coordinates the information encoded by the electrical stimulation and the output decoded from the recorded signals so as to establish an initial force field structure as a map between the position of the device and the force applied to it. We should stress that this approach does not need to be limited to represent a static field. Instead, the force may be expressed as a function of velocity, acceleration or any combination of state variables that are encoded for example by motor cortical activity [22]. Here we focus on static fields for computational simplicity. The force field structure, being encoded in the stimulus-response relation of a neuronal population, can then be modulated by brain activities, including volitional commands impinging upon the recorded neurons from other brain regions. Indeed the desired behavior of this bidirectional interface is similar to the one exhibited by spinal and supraspinal reflex mechanisms that, at the same time, permit to the brain to modulate the force field by generating new families of trajectories.

In the following, we first describe in detail the algorithms that we developed for calibrating the encoding and decoding components of the interface to approximate non-linear desired force fields and for letting the ndBMI evolve to control a simple simulated mechanical system. We then test and validate the ndBMI using neural activity of populations of neurons in a simulated sensory-motor cortical system. We analyze how this interface approximates a desired dynamical behavior associated with non-linear field acting upon a simple mechanical system. We consider two force fields: a radially convergent force field used to represent reaching tasks and a dipole force field used for representing obstacle avoidance in manipulation and navigation tasks. We evaluate the performance of the ndBMI in generating trajectories by simulating different kinds of configurations of the stimulating and recording electrodes. Finally, we explore the computational issue of controlling the operation of this interface by volitional commands.

## Materials and Methods

**Figure 1:**
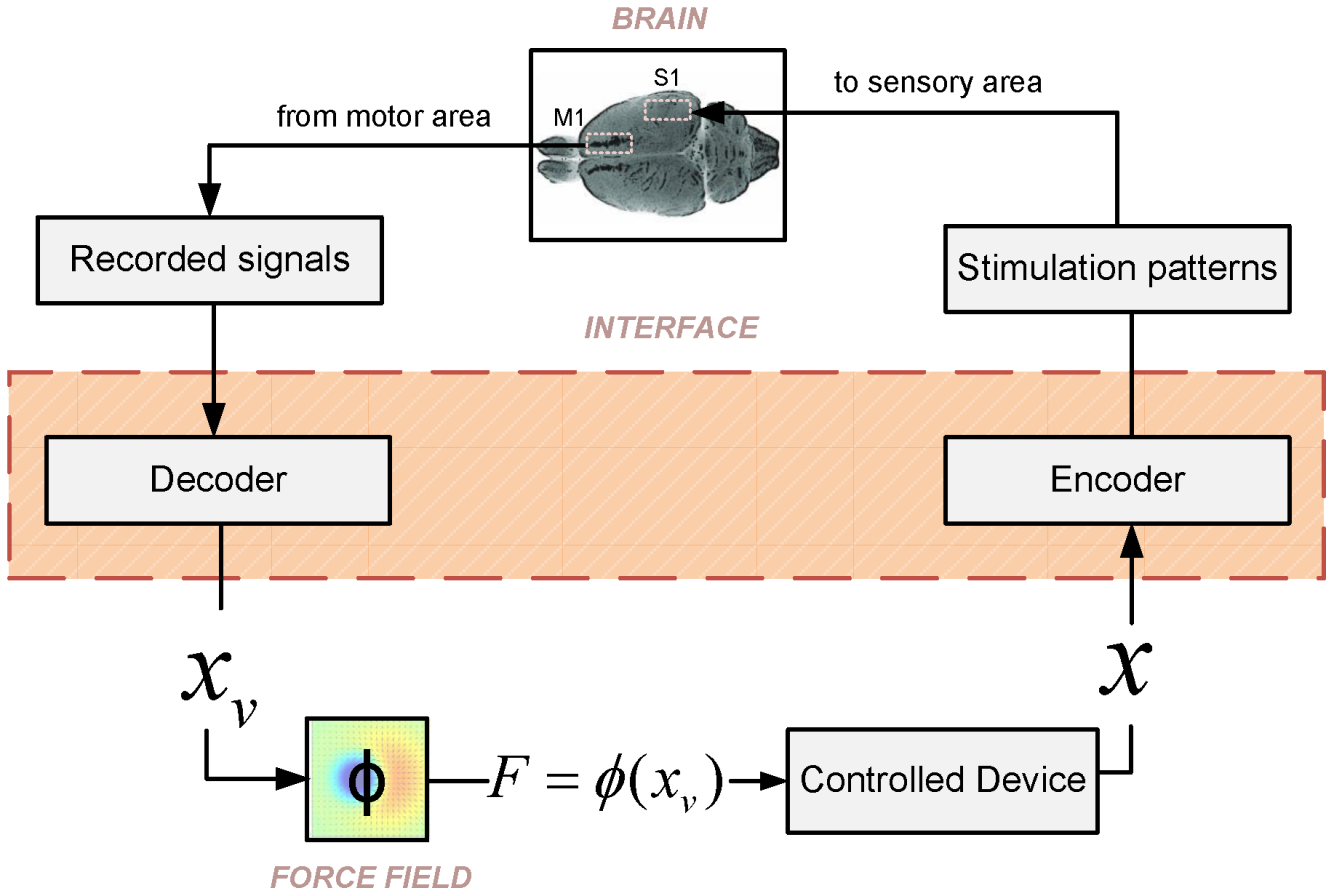
Schematic of the closed-loop non-linear dynamic (ndBMI) Brain-machine Interface. The neural signal recorded from a motor region of the brain is decoded by the motor interface into a point in the position-space of the controlled device. The force 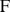 to be applied to the external device is computed by first mapping the recorded motor cortical activity into a virtual point 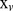 in the position space of the device. This virtual point corresponds to the most likely position of the device given the observation of the activity of the motor cortical region. Then, the force 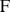 is derived by calculating the desired force field in this virtual point 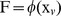. Once this force is applied, the device evolves to the next position 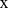. The sensory interface provides information to the brain about the new position of the device by delivering stimulation to a sensory area. Once it receives this information, the brain generates a consequent motor cortical response which is again translated into a force, in a closed loop.

We begin by summarizing the structure of our ndBMI, which is illustrated in Figure 1. The ndBMI controls an external device (in our case a simple simulated mechanical system) and is constructed by closing the loop between two components: the *sensory interface* and the *motor interface*. The sensory interface maps some or all of the state parameters of the external device (in our case only the position of the device) into one of a set of possible patterns of electrical stimulation delivered to a cortical sensory area. The result of this operation is that the activity evoked in this sensory area is made to encode the state parameters of the device. The motor interface takes neural recordings from a motor cortical region and translates them into a force applied to the object. This force 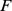 is applied to the device which then evolves to the next state. The brain is informed about the value of the next state by the sensory interface and generates a new appropriate motor cortical response, and so on in a closed loop.

The algorithms calibrate the interface so that, in absence of any voluntary intention to change the behavior, the interface approximates a given desired force field (i.e. a function 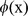 expressing the force that we would apply to the device when it is in state 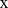). In the force fields implemented here, this force was designed to drive the device toward an equilibrium state. The fact that the force to be applied is decoded from the actual neural activity (and not only directly from the desired force field) leaves open the possibility to modulate at will the actions implemented by the ndBMI (for example to deliberately shift the position of the equilibrium point of the device) when the brain expresses additional voluntary components of motor cortical activity.

The algorithms base both the conversion of information about the state of the device into brain activity elicited by stimulation and the decoding of motor cortical activity into a set of forces needed to accomplish the task on an explicit map between spike trains and the state space of the device computed with Multi-Dimensional-Scaling (MDS).

The neural data that are used to construct and run the ndBMI consist of stimulus-response pairs of a given electrical stimulation pattern applied in sensory cortex and the associated neural population response recorded in motor cortex. They are divided into two groups: calibration data, which serve to set the parameters of the sensory and motor interfaces so that they approximate the desired force field, and test data, which serve to operate the interface and test its ability to control the considered external device.

In the following we report a detailed description of the algorithms for calibrating and running the interface, and of the external mechanical system controlled by the interface. We developed and tested this interface by using synthetic neural responses generated by simple descriptive model of cortical responses of a cortical motor region (here supposed to be the part of the primary motor cortex (*M1*) that controls whisking in rats) following a simulated electrical stimulation applied to the whisker region of primary somatosensory cortex (*S1*). These simulations, which we also describe in the following sections, were chosen to mimic this particular sensory-motor cortical system because this is the one we used in previous experimental work on linear bidirectional interfaces [21].

### Summary of the Algorithmic Function of the Interface

The mechanical system that the interface controls is simply a point mass moving on a plane towards a target equilibrium region (see section *Simulation of the mechanical system used as external device*). This target region is defined as a zone around the equilibrium point of the approximated force fields, whose dimension is a parameter of the simulation. We thus start each run of the operation of the interface by setting the system at an initial position 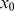 with zero velocity. From this initial position, the interface simulation algorithm proceeds as follows.

First, the sensory interface computes the electrical stimulus 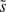 corresponding to the sensory region that contains the current position 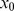 of the external device using the following equation:
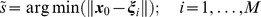
(1)where 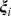 is the center of the sensory region 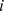, 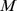 is the number of the stimulation patterns (therefore is also the number of sensory regions), and 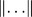 is the Euclidean distance norm on the device’s position plane.

The interface applies the electrical stimulus 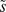 to the sensory cortical area and records a response 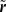 from the motor cortical area. The motor interface derives, from the motor cortical responses 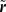, the force 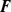 using the algorithm described in section *Calibration of the motor interface*.

The interface applies the force 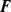 to the mechanical system and lets it evolve for a fixed amount of time until it reaches the next position 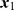 as described in section *Simulation of the mechanical system used as external device*. Between successive stimuli, the force (i.e. the output of the interface) is assumed to remain constant.

The procedure is repeated until the point mass reaches the target region.

The task is considered to be completed when the point mass reaches the target region (this situation is termed a “convergent” trajectory) or when the number of iterations of the process reaches a maximum value of 50 iterations (we termed this situation a “non-convergent” trajectory). After the task is completed, we start the process again by starting the evolution of the system at rest (0 velocity) from another random initial position 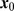.

Operating the ndBMI as described above requires the calibration of the parameters of the sensory and motor interfaces. This is described next.

### Calibration of the Sensory Interface

To calibrate the sensory interface we constructed a map from the position of the external device to a corresponding electrical stimulation. The final product is a partition of the position space of the external device into a set of “sensory regions”, each being associated to a particular electrical stimulus. The calibration of the sensory interface was implemented as follows.

Assume that we applied a set of 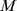 electrical stimuli 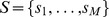. We recorded 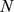 “calibration responses” of each of the 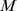 electrical stimuli (total 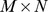 responses):
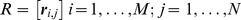
(2)


Each response 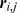 consists of a sequence of spike times for each of the recorded neurons in a simulated 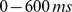 post-stimulation window (we chose this window length because it matched that used in our previous real neurophysiological implementations of a linear bidirectional BMI [21]. However, using such a long response integration window is by no means necessary. We verified on the current algorithm with simulated data (results not shown) and on the previously published linear algorithm by running the interface offline from real cortical spike trains that we recorded [21], it was possible to reach near-maximal performance with response windows as short as 50 ms). We then calculated the matrix of spike train distances between all pairs of neural responses across all stimuli,
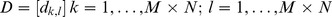
(3)



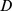
 is a symmetric matrix with zero diagonal and indexes 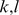 that run over all possible stimulus-response pairs. To compute distances between multiple-neuron spike trains, we used the metric described by Houghton and Sen [23], to which we refer for full details. In brief, this metric first convolves the spike trains in the time domain using exponential kernels (whose decay time constant 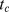 represents the temporal sensitivity of the metric and is a free parameter of the analysis) to obtain the response time vector of each individual neuron. It then computes the multi-neuron spike train distance using a vector norm computed after rotating these single neuron vectors by an angular free parameter 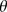 that determines the sensitivity of the metric to neuron-to-neuron differences [23]. The free parameter 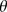 can vary between 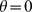 (corresponding to pooling all spike train without taking into account the identity of which neuron fired which spike) and 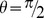 (corresponding to considering a labeled-line code [24], [25] in which the identity of which neuron fired each spike is fully taken into account). In the analysis presented here, we set the two free parameters of the metric to 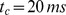 and 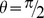 because we found that these parameters empirically maximized the performance of the interfaces.

We used MDS to construct a system of points in the position domain of the mechanical system (in this case it was a 2-dimensional domain) that preserves the spike train distances:
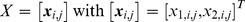
(4)


Where 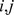 runs over the indexes of the 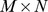 calibration responses. To perform the non-metric multidimensional scaling operation, we used the “*mdscale*” function in MATLAB by choosing the metric scaling “*strain*” as the goodness-of-fit criterion to minimize, which is a criterion equivalent to that used in classical MDS.

We multiplied these vectors of positions by a factor 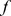 to make them fit within a box of the size of the position space:
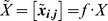
(5)


Finally we computed the averaged positions across calibration trials to each given stimulus pattern to obtain the so called “calibration site”:
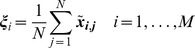
(6)


These 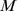 calibration sites were then used to partition the position space into 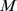 sensory regions, by associating each point of the position space to the nearest calibration site 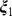 according to Equation 1.

### Calibration of the Motor Interface

The purpose of the motor interface is to decode each neural response of the test data set into a force vector. We did this in two steps: first, the motor neural response 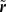 recorded in the considered test trial (the one to be converted into a force) was mapped (by a function that we called 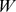 and whose computation is explained below) to a virtual point 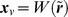 in the position space of the device. This virtual point corresponds to the most likely position of the device given the observation of the activity of the motor cortical region. Then, the force to be applied to the device was derived by calculating the desired force field in this virtual point.

If 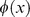 is the desired force field to be approximated by the interface, then the motor interface derives the output force to be applied to the external device as 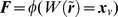. We call the point 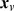 “virtual” because it is not necessarily reached by the point mass (it is, in principle, different from the actual position of the controlled device). This virtual point is only used as an intermediate step to evaluate the force intended by motor cortical activity. Any voluntary perturbation or addition of neural activity, intended for example to shift the equilibrium target point of the controlled external device, will act by shifting the position of this virtual point so as to create the perturbation in force necessary to modulate the behavior according to the volitional command (see section *Addition of volitional control to shift at will the position of the target region*).

We implemented two slightly different algorithms that translate the current test response 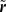 into a force. Both algorithms begin with measuring (using the spike train metric described above) the distances between the currently recorded spike train 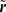 and all the responses in the calibration trials. These distances are stored in a matrix 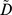:
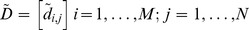
(7)


The algorithms are detailed as follows:

#### *Single-point decoding algorithm*

For each stimulus we computed 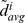, that is the average (across calibration trials to a given stimulus) of the distances between the current spike train 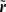 and the spike trains obtained during calibration (the complete separation between the set of calibration and test trials has the purpose of preventing over-fitting). Following [23], [26] the averaging over calibration trials to a given stimulus was performed with a bias exponent of 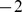 that under-weights outliers:
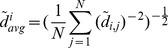
(8)


We decoded the stimulus that evoked the recorded spike train 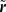 as the stimulus 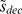 whose calibration responses gave the smallest average distance with the current spike train 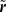:
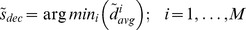
(9)


The force vector applied to the external device (i.e. the dynamical system) was computed as the force value given by the considered force field at the location of the calibration site corresponding to the decoded stimulus:
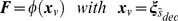
(10)


#### *Multiple-points decoding algorithm*

We first identified the smallest spike-train distance from the current spike train 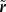.
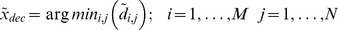
(11)


We then computed the force vector 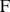 using the force field equation at the location 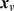 where the closest calibration trial is projected on the domain of the external device by the MDS projection:
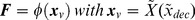
(12)


The advantage of the Single-point algorithm is its robustness to outliers. The appeal of the Multiple-points decoding algorithm is that its repertoire of forces is not limited only to the values of the desired field at the center of each sensory region, but instead it takes full advantage of the variability associated with individual calibration activities to offer a larger spread of output forces and a potentially richer interface dynamics. However, as we shall see in *Results*, both algorithms gave nearly identical performance on the simulated data used here.

### Simulation of the Mechanical System used as External Device

The external device controlled by the ndBMI is a simple simulated mechanical device, i.e. a simulated point-mass moving within a viscous fluid. The point mass is subject to two forces: the force derived from the neural activity 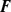 and a drag force due to the viscosity 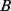:
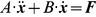
(13)


In the above equations, 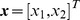 indicates the position of the point mass on a plane and the values of mass 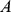 and viscosity 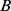 were set to 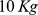 and 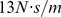 , respectively. We simulated this dynamics equation for a period of 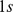 using standard numerical integration algorithms, see [21] for details.

### The Force Field

The calibration procedure establishes a force field as a relation between the position of the device and the force applied to it in the absence of external volitional commands. Because of the stochastic character of the neural responses to the electrical stimuli, the field is an approximation of a desired position-to-force mapping.

The force field established by the calibration procedure is effectively a biomechanical “platform” upon which influences of the environment and of the volitional commands operate to shape the actual motion of the controlled device. In addition to the volitional and environmental influences, the motion of the device is also affected by neural noise. Therefore the state of motion of the device is effectively a random variable affected by a combination of deterministic and stochastic processes. The challenge for the volitional commands is to guide the device to the desired goals despite the influences of uncontrolled perturbing forces.

**Figure 2:**
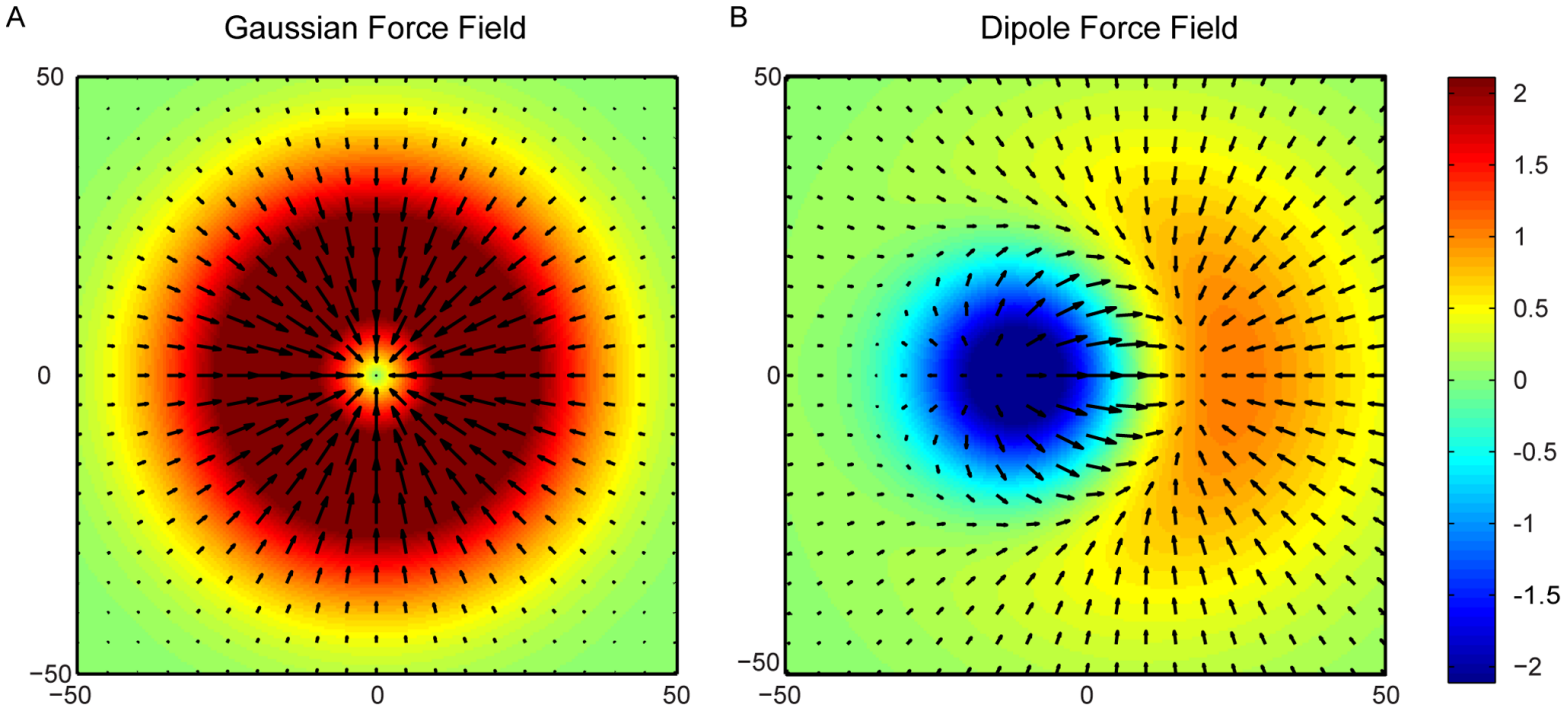
The two ideal force fields to be approximated by the ndBMI. The force fields map each point belonging to the position space into a force. In this study we used two different force fields representing the desired control policies: a Gaussian force field (A) and a Dipole force field (B). Both the force fields converge towards an equilibrium point that represents the goal in a reaching task. The figure shows the force fields represented by arrows of different lengths superimposed by the corresponding colored-code potential fields.

Here, we consider two different types of force field that are significant for the generation of motor behaviors: a Gaussian force field 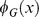 and a Dipole force field 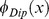 (see Figure 2).

In a Gaussian force field the forces converge toward a central equilibrium point implementing the concept of a single attractor and of the goal of reaching a fixed position.

This convergent field 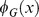 is the gradient of a Gaussian bivariate function [27]:
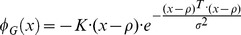
(14)with 
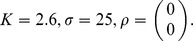


A Dipole force field 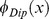 is obtained as a linear summation of a Gaussian force field 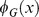 with a Divergent force field 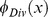:
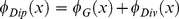
(15)


The divergent field 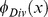 is obtained by summing a repulsive and an attractive field, and has the expression:
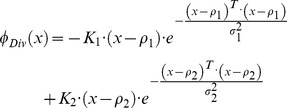
(16)with
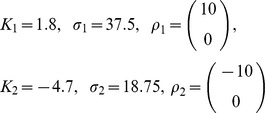



Note that both fields are non-linear and non-invertible (i.e. each value of force is generated at multiple points in the domain). A Dipole force field was introduced in robotics to represent movement planning in the presence of obstacles [28]. The repulsive forces of the divergent field are centered on an object that must be avoided by the moving point mass while the attractive forces are centered on the target to reach.

### Simulated Neural Data

Our ndBMI is designed to encode information about the state of the device by electrical stimulation of a sensory area and to use, in a closed loop, recordings of neural activity in motor cortex to drive the external device. Testing our ndBMI algorithm on synthetic data thus required simulating neural responses in a motor area immediately following electrical stimulation in a sensory area. We therefore simulated spiking responses of neural populations from the primary whiskers motor cortex 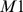 of rats in response to electrical microstimulation of the whisker “barrel” field of primary somatosensory cortex. This model is based on the empirical observation that a focal activation of 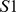 causes a relatively localized activation of 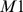 [21], [29], [30]. While our model simulates in a simplified way the net effect of 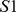 activation on 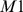 responses, it is a descriptive model which does not make any specific assumption about the mechanisms generating these responses.

**Figure 3:**
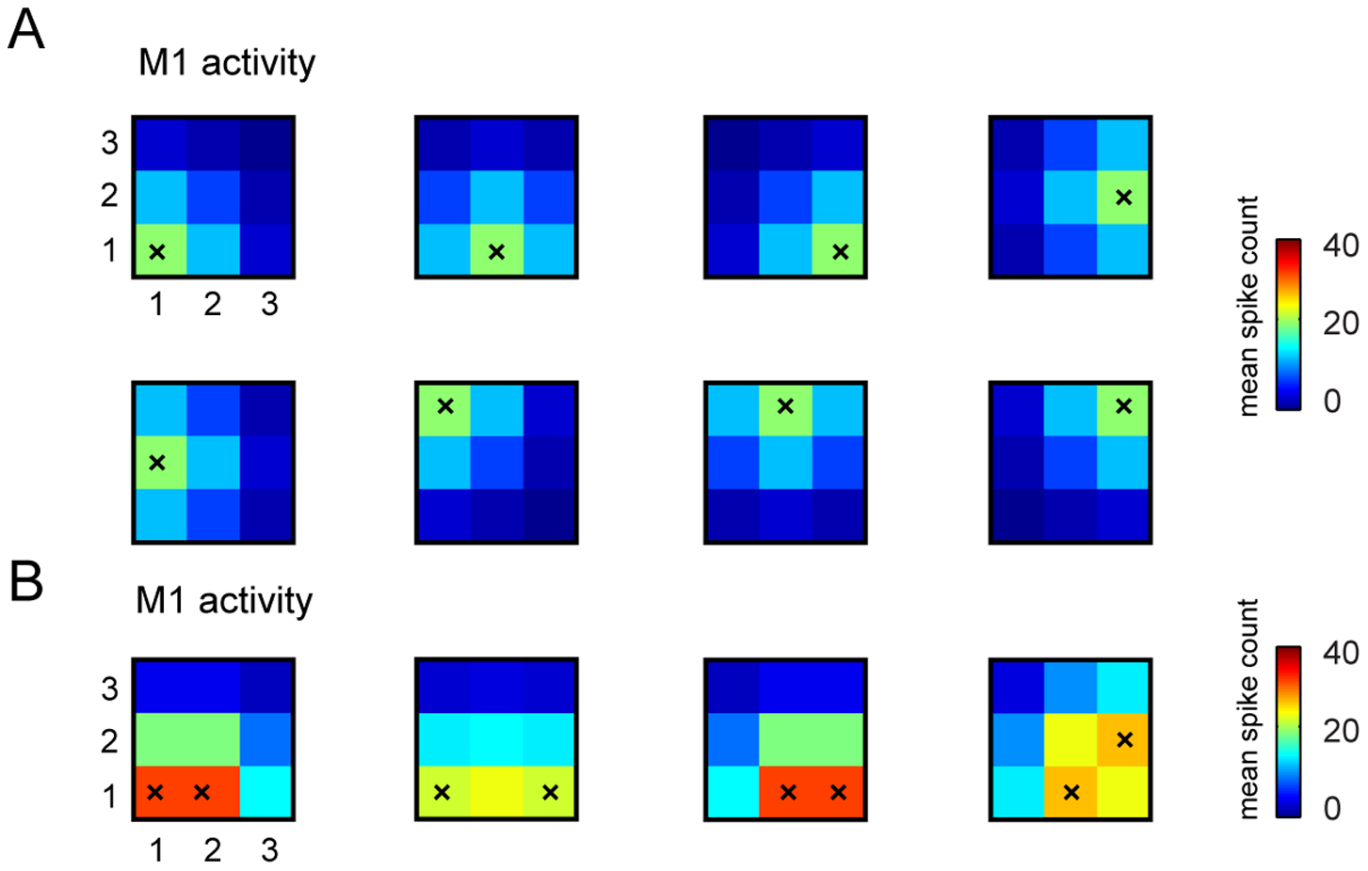
Examples of stimulus-response S1-M1 model used to run the interface. We report some examples of trial-averaged neural responses by our model M1 in response to selected patterns of stimulations of S1. Here we plot responses for the case in which recording electrodes are arranged in a 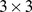 grid (as in the stimulus set number 6 that has 32 elements (see Table 1) and was used in most of the simulations). As explained in the main text, the spikes evoked by stimulating each S1 electrode are modeled by bivariate Gaussian distributions that peak in the corresponding M1 recording sites. (A) The average number of simulated spikes recorded from the electrodes in M1 and evoked by stimulating a single electrode in S1 (stimulated electrode is represented by a superimposed black “x”). (B) Average number of simulated M1 spikes evoked by stimulating couples of electrodes in S1 (again the pair of stimulated electrodes is indicated by the superimposed black “x”). In both panels, the color scale indicates the mean spike count expressed in units of mean spike count per trial, and the responses were shown for only one of the possible four levels of intensity in which each electrode could be stimulated in stimulus set number 6 (see Table 1).

In the model, we assumed that the stimulation and recording electrodes are organized into two different arrays placed in 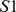 and 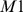 and arranged in a square matrix with the same shape and number in each of the two regions. We assumed that the electrical activation of each stimulation sites in 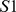, at a given electrical intensity, evoked a certain average number of spikes per trial on each electrode in 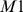. The average number of spikes of each 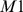 electrode in response to each electrical stimulus was modeled by a bivariate Gaussian with parameters height 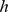 (expressed in units of average spikes per trial, and representing in an abstract way the “intensity” of the stimulation) that peaks in the corresponding 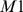 recording site (Figure 3) and spatial spread 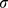 (in units of inter-electrode distance among recording electrodes). The topography of the position of the centers of activation in simulated responses from 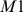 to different stimuli in 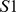 matched that of the simulated stimulating electrodes in 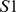. By indexing with 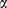 and 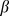 the electrode positions in the horizontal and vertical directions respectively (both for stimulating and recording array, that in our model have the same geometry), the spike rate at each electrode 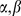 following activation of a given stimulation site *s* indexed by 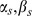 can be thus be expressed as follows:
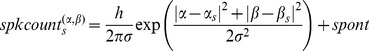
(17)where 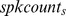 is a matrix with the same dimensions as the electrode array expressing the trial-averaged spike count output (in units of spikes/trial in the response time window used to run the interface). The term *spont* denotes the amount of spontaneous (not stimulus-induced) firing and was set to zero unless otherwise stated.

The topographic stimulus-response arrangement implemented in our simulations is observed to some extent in real data [29] and is useful for visualizing the results of our algorithms. It is important to note that the assumption that the topography of stimulation in the sensory area is preserved by the activity of the motor area is not crucial to the function of the algorithm. The algorithm does not require this assumption because it computes the spatial configurations in the force field space on the basis of the distances between neural activities elicited in different conditions rather than from distances on the cortical surface.

Unless otherwise stated, the spike trains were generated, for each pair of stimulus and recording sites, by drawing randomly interspike intervals from an exponential Poisson distribution of interspike intervals with a mean equal to the average number of spikes recorded in that electrode in response to that stimulus. As a consequence, the spike times are distributed with a Poisson distribution with time-independent firing rates.

**Figure 4:**
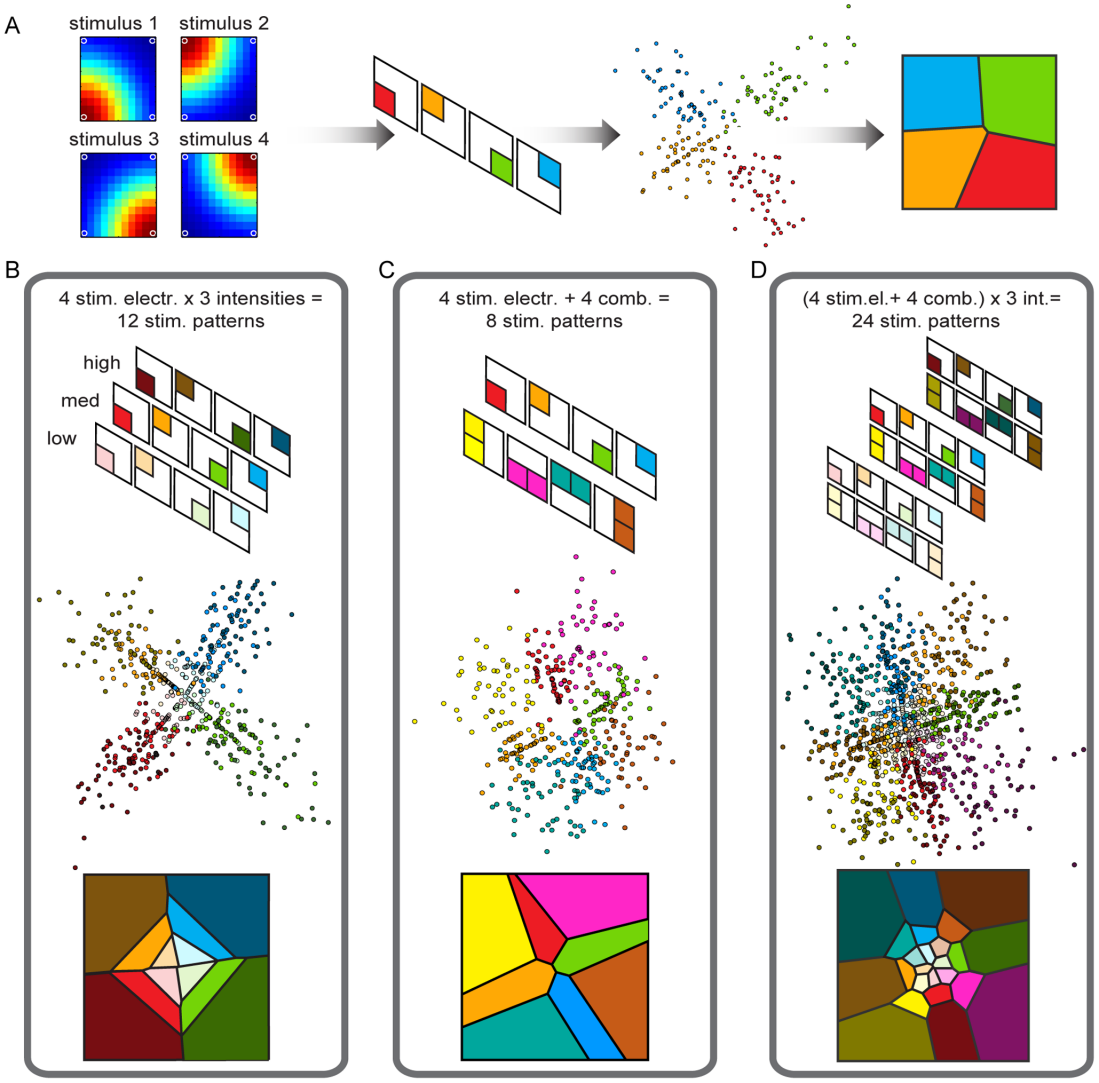
Results of the calibration procedure to set-up the Sensory Interface using different data sets. The goal of the calibration procedure is to define the “sensory regions” by partitioning the position space of the controlled device. (A) from left to right: i) a representation of the evoked spike activity, ii) a color-coded scheme of the recorded spike activity, iii) a graphical representation in the 2D domain of the results of the multidimensional scaling of the distance metric for each spike train, iv) the sensory regions obtained using a nearest neighbor algorithm. We tested this procedure also by using three different stimulation intensities (B), a combination of the stimulation electrodes obtained by co-stimulating neighboring electrodes (C) and by combining the co-stimulation with three stimulus intensities (D).

In our simulations, we considered seven different sets of electrical stimuli used to encode information in the sensory area. The different stimulus sets are obtained by varying, from one stimulus to the next, the number and location of the stimulated electrodes, as well as the intensity of stimulation. Stimulations of multiple electrodes are simulated as sum of responses that would have been evoked by stimulating each electrode individually. The different stimulus set used to generate each data set are summarized in Table 1 and also sketched in Figures 4. Investigating the behavior of the ndBMI when using stimulus sets with such different information characteristics, is useful to better understand how the sensory interface works.

**Table 1:** Schematic description of the stimulus set used to generate each response data set.

Dataset	Number ofpatterns	Description	σ	Peak responseamplitude (h)	f factor	Electrodesgrid
1	4	In each stimulus, each of four different stimulussites is activated with one intensity level.	0.5	5	[35.24–37.85]	2×2
2	12	Each stimulus site is activated at one of three intensity levels.	0.5	2–5–8	[21.61–27.54]	2×2
3	8	Stimulus sites are activated either individually orin contiguous pairs at one intensity level.	0.5	5	[23.08–21.66]	2×2
4	24	Stimulus sites are activated individually orin contiguous pairs at three intensity levels.	0.5	2–5–8	[15.79–19.70]	2×2
5	2^3^ = 8	4 stimulation electrodes × 2 electrical intensities	0.5	5–10	[8.27–6.85]	2×2
6	2^5^ = 32	8 stimulation electrodes × 4 electrical intensities	1	10–20–30–40	[2.94–2.85]	3×3
7	2^7^ = 128	16 stimulation electrodes × 8 electrical intensities	1	10–20–30–40–50–60–70–80	[1.25–1.31]	5×5

For each data set (1 to 7) we describe the stimulus set, we report the parameters spatial spread (σ), peak response amplitude 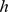 (reported in units of mean spike count per trial and varied to model the different amplitudes of stimulation), the value of the scaling factor 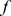 and the geometry of the grid of both stimulated and recording electrodes. We use square grids of electrodes, but for stimulation we only use the “external” sites, located on the perimeter of the array. The recording electrodes are all used. So for data set 5, the matrix of electrodes is 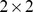 and we use all of them. For data set 6, the grid is 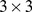, and since we do not stimulate with the central one, we use 8 stimulation electrodes, while all 9 recording electrodes are used. The grid used in data set 7 is 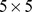 and we do not stimulate with the 9 internal electrodes.

We note that the above model of neural firing, as well as the above algorithm that we developed for the interface, assumes a stable relationship between stimulation of S1 and activity evoked in M1. However, in later Sections we investigate by numerical simulations both how the interface behaves when the S1-M1 map is changed by voluntary perturbations (Section “Addition of volitional control to shift at will the position of the target region”) and when the map between S1 stimulation and M1 recorded responses is altered after calibration by a deterioration of the selectivity and quality of the recorded neural responses (Sections “Deteriorating the simulated quality of neural responses to investigate the robustness of the algorithm” and “Robustness analysis of the ndBMI system”).

### Deteriorating the Simulated Quality of Neural Responses to Investigate the Robustness of the Algorithm

To evaluate the robustness of the ndBMI to degradation of the quality of responses, we generated neural activities with different amount of information about the electrical stimuli. We used several different ways to deteriorate such responses.

The first alteration of the quality of neural responses decreases the information about stimuli available at one or more electrodes. This was achieved by “flattening” the stimulus-to-stimulus variations in trial-averaged spike count (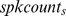) to each stimulus 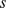 without changing the overall averaged spike count in response to all stimuli 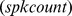, as follows [31]:
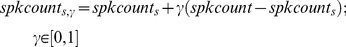
(18)


For 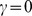 the spike counts are equal to the original ones and all original stimulus information is available, while for 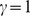, each stimulus triggers the same spike count and information is zero.

The second simulated alteration of the neural responses tested the effect of the statistics of neural firing and consisted in generating spike trains with a Gamma distribution of interspike intervals (rather than with an exponential distribution like for the Poisson process), as follows:
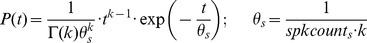
(19)


This distribution fits cortical interspike interval distributions well [32]. For any value of the so called shape parameter 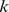, it produces a mean inter spike interval equal to 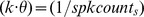 with an amount of trial-to-trial spike count variance that depends on 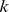. The case 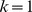 corresponds to the Poisson process (variance equal to mean), whereas values of 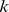 lower than (respectively higher than) one generate trains with a higher (respectively lower) variance than the one of the Poisson process. Studying the performance of the algorithm as a function of the shape parameter 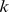 therefore allows us to investigate the specific role of neural variability in the interface.

The third simulated alteration of neural responses tested the effect of spontaneous activity upon the performance of the interface. This was achieve by setting to a non-zero value the term 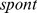 of spontaneous firing in Eq. 17.

The fourth simulated deterioration consisted in simulating a “misplaced” recording electrode (of coordinates 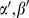) unable to record a response modulated by the simulation (this can happen for example, because the recording electrode is placed outside the region modulated by the stimulus, or because the electrode is highly corrupted by noise). The average spike count recorded in the misplaced recording electrode topographically matched to the considered stimulation electrode was set to be constant across all stimuli (and equal to the grand average number of spikes recorded across all electrodes and stimuli) and was expressed by the following equation:
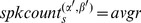
(20)for each possible stimulus 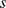, where 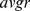 is the grand average number of spikes recorded across all electrodes and stimuli.

The fifth simulated deterioration mimicked a situation in which a stimulation site 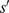 was made ineffective by triggering uniform responses across all recording sites (again equal to the grand average number of spikes recorded across all sites and stimuli), as follows:
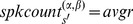
(21)for each possible electrode position 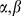.

Since the responses generated by stimulating this electrode do not have any spatial specificity and do not change with stimulus intensity, this electrode encodes no information.

### Quantification of the Trajectories and Performance Evaluation

For each simulation, we started by placing the point mass at an initial position 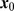. Then we let the interface run for up to 50 time steps. We randomly chose 24 different initial positions along a square centered in the origin with side equal to 0.8 times the dimension of the position space and for each of these 24 positions, we performed 10 repetitions, obtaining a total of 240 trials.

To measure how well our data sets and algorithms approximate the ideal force field, we introduce a metric called *within-trajectory position error* (abbreviated to *wtpe*) that measures the average distance, across all time steps, between each convergent trajectory (as defined in section *Summary of the algorithmic function of the interface*) and the ideal trajectory obtained by simulating the mechanical system under the influence of the desired force field and in the absence of noise. If a trajectory goes from time step 1 to 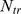 with 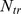 indicating the number of steps needed to converge for a given trial 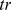, 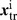 is the position of the point mass at time step 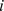 for a given trial 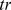, and 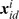 is the position of the point mass at that time step in the ideal force field, then *wtpe* is the averaged error:
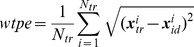
(22)


High (respectively low) values of *wtpe* denote bad (respectively good) convergence performance of the system.

Significance of variations in *wtpe* were assessed using one-way ANOVA (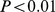) followed by the comparison between a reference condition and all the others using a multiple comparison test with Tukey’s honestly significant difference criterion based on the Studentized range distribution.

## Results

We used the simulated cortical responses to illustrate the behavior of the ndBMI and investigate its performance in a number of different conditions.

### Calibration of the Sensory Interface on Simulated Data: The Sensory Interface Captures the Geometry of the Simulated Motor Cortical Activity Evoked by Stimulation of Sensory Cortex

The first step to set up the ndBMI is the calibration of the sensory interface using the training set (i.e. calibration data) of simulated neural data. In this work we generated the training set using 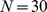 trials for each stimulation pattern. This process defines the 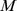 regions of space corresponding to each stimulation pattern 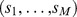 by projecting (using *MDS*) motor cortical responses into the position plane in a way that preserves the original spike train distances of neural responses to different stimuli.

To illustrate the relationship between the spatial distribution of the information encoded by electrical stimulation and the geometry of the sensory regions, we first ran the calibration procedure using 4 different sets of simulated spike trains (Table 1 and Figure 4), each with a different stimulation geometry. The corresponding sensory interfaces and the partitions of the position space of the external device are reported in Figure 4.

Note that these first 4 data sets used to illustrate the properties of the sensory interface are slightly different from those used in the next sections to test the behavior of the ndBMI, but we chose to start from these 4 data sets because they illustrate very clearly the relationships between information encoded by stimulation and the partition into sensory regions made by the interface.

Data set 1 was generated by using a simple configuration, in which each stimulation pattern was constituted by the activation of only one of the stimulating electrodes. In this case, the sensory interface produced 4 well-separated regions that represent very well the geometry of the clusters of the 2D-projected spike trains (Figure 4A middle). The positions of the calibration sites are well-spread over the work space along two orthogonal lines obtaining similar-sized sensory regions (Figure 4A right).

We then considered a second data set that used the same single-electrode stimulation patterns of the first data set, but that in addition presented three different intensity stimulation levels (low, medium and high). This stimulus set had 12 different stimuli and, as a consequence, there were 12 sensory regions. The addition of the stimulus amplitude variable that modulated the response in the motor region changed the geometry of the sensory regions in several interesting ways (Figure 4B). The increase of the evoked spike activity with stimulation was encoded as an increment of the distance from the center of the workspace along the two main diagonals. The algorithm generated sensory regions that were organized such that increasing stimulation intensities resulted in increasing distances from the center. Thus, while the position of the electrode was encoded as an angle from the origin of the plane, the amplitude of stimulation was encoded as a radial distance from the origin.

To investigate the effect on the geometry of the sensory interface of inserting more complex stimulation patterns into the stimulus set, we created a data set with new stimuli made with the simultaneous stimulation of neighboring electrodes (data set 3). This resulted in a total of 8 distinct stimulus patterns (Figure 4C). In paired stimulations, we assumed that the same current pattern was simultaneously delivered through two neighboring electrodes. For each combination of two electrodes, the evoked spike trains were projected by the algorithm into the portions of space left by the projections of the spike trains evoked by each single electrode of the couple. Thus, the spatial configuration of the sensory regions reflected the combination of the electrodes in the stimulating array.

We finally tested the algorithms by using all the 24 different stimulation patterns described above (data set 4). The generated sensory regions reflected the spatial configuration of the electrodes and the intensity of the stimulation patterns (Figure 4D). For simplicity in the following sections we will run the interface using only stimulus set made of individual electrode stimulations.

### Non-linear Dynamic BMI System: Dependence of Performance upon the Density of Stimulation Patterns

We next tested the dynamical behavior of the ndBMI and its ability to control and interact with the external device, by first constructing the sensory and motor interfaces as described above, and then evaluating the trajectories of the point mass controlled by the ndBMI on a separate test dataset.

We tested two different ndBMIs that implemented two types of desired control policy of the external object and corresponding to two different force fields. The first was a Gaussian force field with all the forces converging toward a single equilibrium point (Figure 2A). The second was a Dipole force field obtained by linear superposition of the previous force field with a divergent one (Figure 2B).

**Figure 5:**
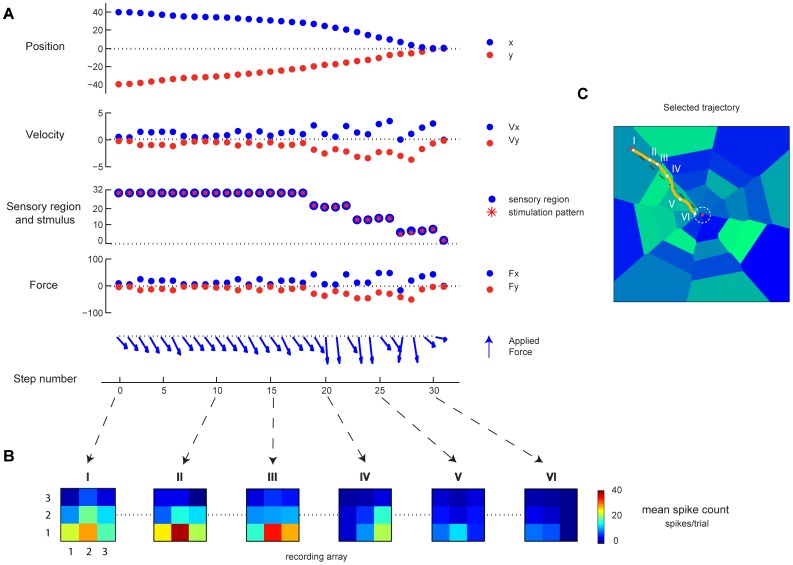
Evolution of variables of the ndBMI system for a single simulated trajectory. (A) Temporal evolution in terms of simulation steps of variables describing the behavior of the system for a single trajectory: for each step from top to bottom are represented the two components of the actual position and velocity of the simulated point mass, the sensory region where the actuator is, the delivered stimulation pattern and the force applied to the dynamical system. (B) Heat maps describing the recorded neural activity in terms of mean spike count for each recording electrodes in 6 different points of the trajectory. (C) A representation of the selected trajectory with a reference to the 6 points depicted in (B) superimposed to the sensory regions.

We used the test set of simulated neural responses to test the ability of the ndBMI to drive a simulated point-mass moving in a viscous fluid, with the goal of reaching an equilibrium target region (indicated by a white dotted circle) around the center of the field. The performance was evaluated by initially placing the mass at rest in a given location and then following its trajectory along the neurally generated force field. An example of the convergent behavior of the BMI in one single trajectory is shown in Figure 5, where we show how the neural activity, the decoded forces and the stimulation patterns evolve with time to accompany the evolution of the position and velocity of the simulated point mass from a peripheral position to the equilibrium target region.

To evaluate how the behavior of the ndBMI depends on the resolution of the information encoded by electrical stimulation, we evaluated how the performance of the ndBMI is affected by the spatial density and number of stimulus patterns available in the set. We generated three different sets of data (see Table 1) by combining a variable number of stimulating electrodes (i.e. 4, 8 and 16 stimulating electrodes) with different stimulation intensities.

**Figure 6:**
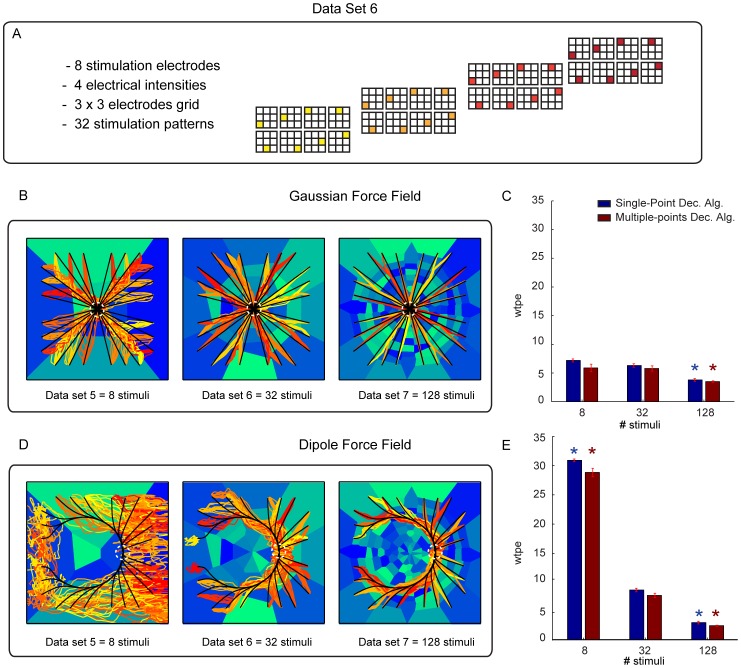
Performance of the ndBMI depends on the resolution of the sensory interface. (A) An illustration of one of the data sets used in this study (data set 6) which is obtained by stimulating 8 electrodes organized in a 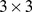 square grid with 4 different electrical amplitudes. (B,D) The actual trajectories (lines colored in red-tonality) obtained with the Multiple-points algorithm and the ideal trajectories (black lines) superimposed to the sensory regions (areas colored in blue-tonality) for a Gaussian force field (top) and Dipole force field (bottom). (C,E) bar chart of the average difference (i.e. error) calculated as the difference between the ideal and the actual trajectory for each trial simulated by using the Single-point (blue) and the Multiple-points (red) decoding algorithm. The * denotes that *wpte* depended significantly on the number of stimuli (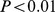 one-way ANOVA) and is placed in correspondence of the number of stimuli for which *wpte* was significantly different from the “reference” condition with 32 stimuli (Tukey multiple comparison test 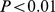).

Figure 6 shows the behavior of the system obtained with a Gaussian force field (middle panel) and with a Dipole force field (bottom panel). As described previously, we first used the calibration data to compute the sensory regions associated with each stimulation pattern. These sensory regions (reported as blue-tonality filled regions in Figure 6) followed a pattern fully consistent with the rules of thumb described in the previous section. We then used the test data to run the interface starting from 24 different initial positions. The trajectory generated by the interface (red-tonality lines in Figure 6) were compared with the “ideal trajectories” (black lines) obtained by simulating the point mass in the exact desired force field (the one that is defined in equation 14 and 15 and that would be generated by the ndBMI in case of infinite spatial resolution of the information encoded by the electrical stimulation and a noiseless neural motor activity). We run the system 10 times from each starting position, thus obtaining 240 trajectories.

We evaluated the interface with both the Single-point and the Multiple-points decoding algorithms for computing the force to be applied to the point mass (see section *Material and Methods*).

The analysis of the performances showed that an increase of the number of the stimulus patterns and, consequently, of the number of sensory regions, made the actual trajectories of the controlled device more similar to the “ideal” trajectories of the desired force field. The advantage of having more sensory regions was more pronounced for the Dipole force field than for the Gaussian force field. For the Gaussian force field there was no gain in increasing the sensory regions from 8 to 32 but the trajectory error decreased significantly when increasing the number of sensory regions to 128 (Figure 6C). For the Dipole force field, there was a significant decrease in the trajectory error both when increasing the sensory regions from 8 to 32 and from 32 to 128 (Figure 6E). This suggests that the gain of having a large number of sensory regions (and thus of patterns of electrical stimulations eliciting different neural responses) is more relevant when implementing more complex force fields and control policies. We also found out that the performance of the system for these simulation conditions was very similar with both the Single-point and the Multiple-point different decoding algorithms.

### Robustness Analysis of the ndBMI System

In a complex system, such as a bidirectional BMI, stability and robustness are crucial features that concern all the elements constituting the information flow, from the recorded electrophysiological signals to the design of the actuators [33]. We tested the robustness of the presented algorithm by investigating its performance in a number of simulated scenarios in which the same degree of degradation of the stimulus-response properties was applied to both calibration and test data.

In all the following tests of the robustness of the interface reported in this subsection, for simplicity we concentrated on the middle-resolution stimulation set (i.e. data set 6, consisting of 32 stimulation patterns). As in the previous section, we run the interface starting from 24 initial positions and computing 10 trajectories of the neurally driven point mass for each of the 24 starting points. Again, we tested the interface with both the Gaussian and the Dipole force fields.

**Figure 7:**
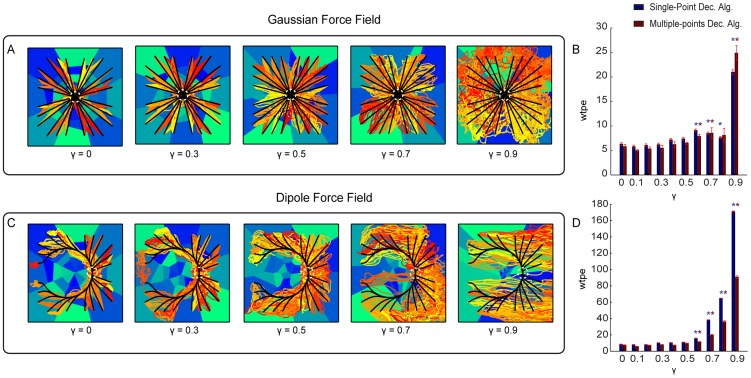
Performances of the ndBMI when reducing information about the stimulation pattern. (A,C) Ideal (black lines) and actual (red-tonality lines) trajectories of the Multiple-points algorithm superimposed to the sensory regions (blue-tonality areas) by using data set 6 and two different force fields simulated by progressively reducing the amount of available information represented by 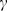, with 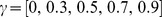. (B,D) Bar chart of the *wpte* between the ideal and actual trajectories calculated for different value of 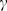. The * denotes that *wpte* depended significantly on 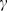 (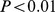 one-way ANOVA) and is placed in correspondence of the values of 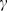 for which *wpte* was significantly different from the “reference” condition with 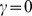 (Tukey hsd 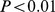).

The first degradation scenario was simulated by progressively reducing the amount of available information about the stimulation pattern that it was possible to extract from the evoked neural response. This degradation was implemented by flattening the different response profiles between stimuli, modulated by the parameter 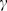 (see section *Deteriorating the simulated quality of neural responses to investigate the robustness of the algorithm*). This parameter, when varied from 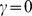 (maximal information) to 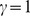 (minimal information) progressively reduced the stimulus modulation of all motor cortical responses. Modulating 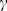 can be thought as modulating the effectiveness of the stimulation and recording electrode implant in eliciting selective responses. Figure 7A,C shows the trajectories collected using the Gaussian and Dipole force fields with 5 different values of 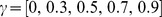. The corresponding mean error between the actual and ideal trajectories for each value of 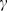 is shown in Figure 7B,D. This analysis shows that both Single-point and Multiple-points algorithms are relatively robust, for both force fields, to the degradation of the response. The mean trajectory error remained relatively stable as 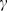 is increased. The interface implementing the Gaussian force field was particularly robust, at its performance deteriorated 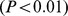 from the reference 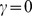 value when 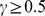 with a small effects and with a large deterioration effects for 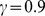 (corresponding to 90% deterioration of response selectivity). The performance of the Dipole force field interface deteriorated significantly 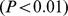 for 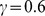 and above, with particularly large deterioration effects reached for 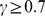.

**Figure 8:**
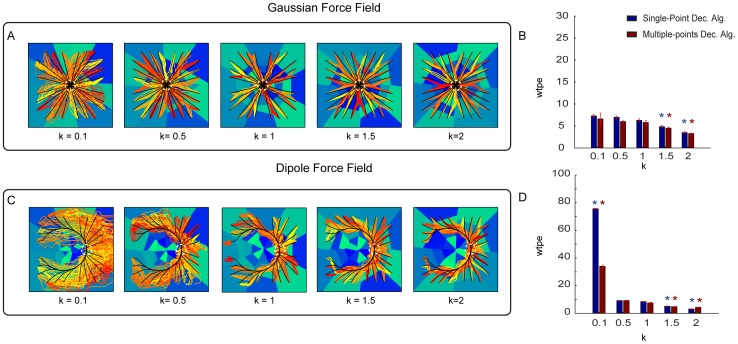
Performances of the ndBMI by using a data set generated from a Gamma Interval process with different values of the shape parameter k. (A, C) Ideal ad actual trajectories superimposed to the sensory regions obtained by using data generated by using five different values of the shape parameter 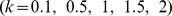 of a Gamma interval process used to set the inter-spike intervals of the simulated spike trains. We ran the interface using the Gaussian (A) and Dipole (C) force fields with the Multiple-points algorithm. (B, D) Average within-trajectory position error across the three different conditions shows how the error decreases by increasing the regularity of the spiking of the M1 neurons. The * denotes that *wpte* depended significantly on 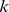 (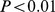 one-way ANOVA) and is placed in correspondence of the values of 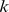 for which *wpte* was significantly different from the “reference” condition with 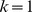 (Tukey hsd 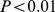).

The second simulated alteration of the neural responses tested the effect of the statistics of neural firing and consisted in generating spike trains with a Gamma distribution of interspike intervals, rather than with a Poisson process as in all the other simulations. The Gamma distribution has a so called “shape” parameter 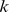 that determines the spike count variance [32]. The case 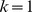 corresponds to the Poisson process (variance equal mean), whereas values of 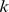 lower than (respectively higher than) one tend to generate trains with a higher (respectively lower) variance than the one of the Poisson process. When implementing the interface on simulated data with different values of 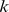 and thus with varying degrees of variance (Figure 8), we found that the performance of the Gaussian force field interface was relatively insensitive to the firing statistics, with significant (P<0.01) deviations from the Poisson case observed for very regular firing statistics (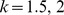; Figure 8A,B). The performance of the Dipole force field interface was more affected by firing statistics, with a particularly marked decrease of performance for processes with 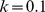 (Figure 8C,D). (Note that values of 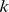 below 0.5 are extremely uncommon in cortical neurons [32]; therefor the value of 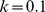 should be interpreted as a case of an extremely irregular firing processes with variability much higher than that of a typical cortical neuron).

**Figure 9:**
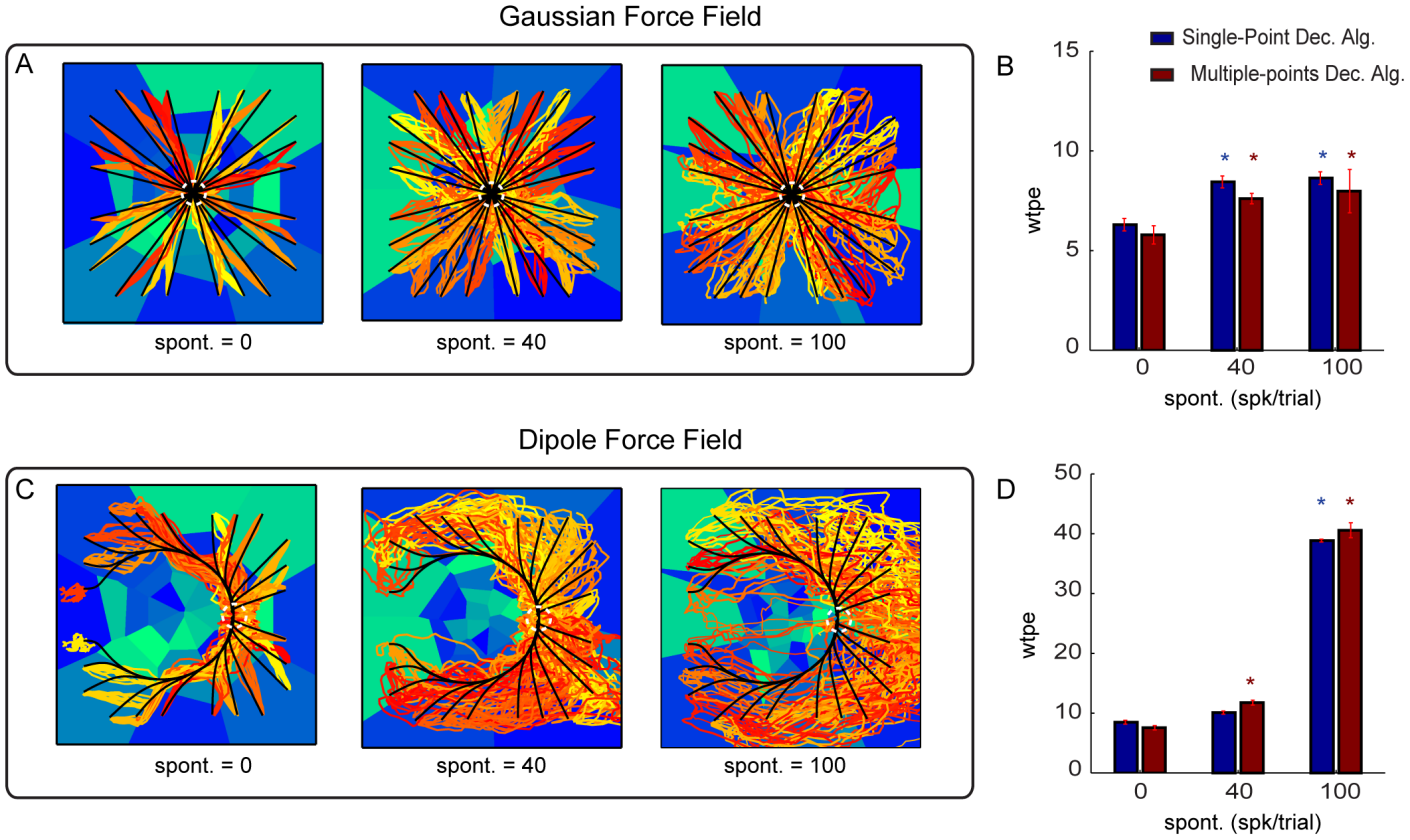
Performances of the ndBMI by adding different level of spontaneous activity to the stimulus-related activity. (A, C) Ideal and actual trajectories superimposed to the sensory regions generated by adding to the stimulus-evoked activity of data set 6, two different levels of spontaneous activity in terms of trial-averaged spike count (i.e. 40 and 100 spikes/trial). We tested the interface by using the Gaussian force field and the Dipole force field with the Multiple-points decoding algorithm. (B, D) Average within-trajectory position error across the 3 different levels of added spontaneous activity tested with the two different decoding algorithms. The * denotes that *wpte* depended significantly on 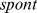 (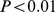 one-way ANOVA) and is placed in correspondence of the values of 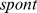 for which *wpte* was significantly different from the “reference” condition with 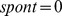 (Tukey hsd 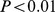).

The third simulated alteration of neural responses tested the effect of adding spontaneous firing. We found that the Gaussian force field interface performance was again extremely robust to this alteration. It decreased only by a relatively small amount (less than 20%) even when adding a level of spontaneous activity that was more than twice larger than the peak response to the maximal simulated electrical stimulation amplitude (this stimulus provoked a peak activity of 40 spikes/trial in these simulations). The Dipole force field interface was also relatively robust to introduction of spontaneous activity of 40 spikes/trial (as large as the peak of the increase of response to the most effective stimulus, Figure 9C,D) but was less robust than the Gaussian force field to values of spontaneous activity much larger than the peak response (Figure 9A,B).

**Figure 10:**
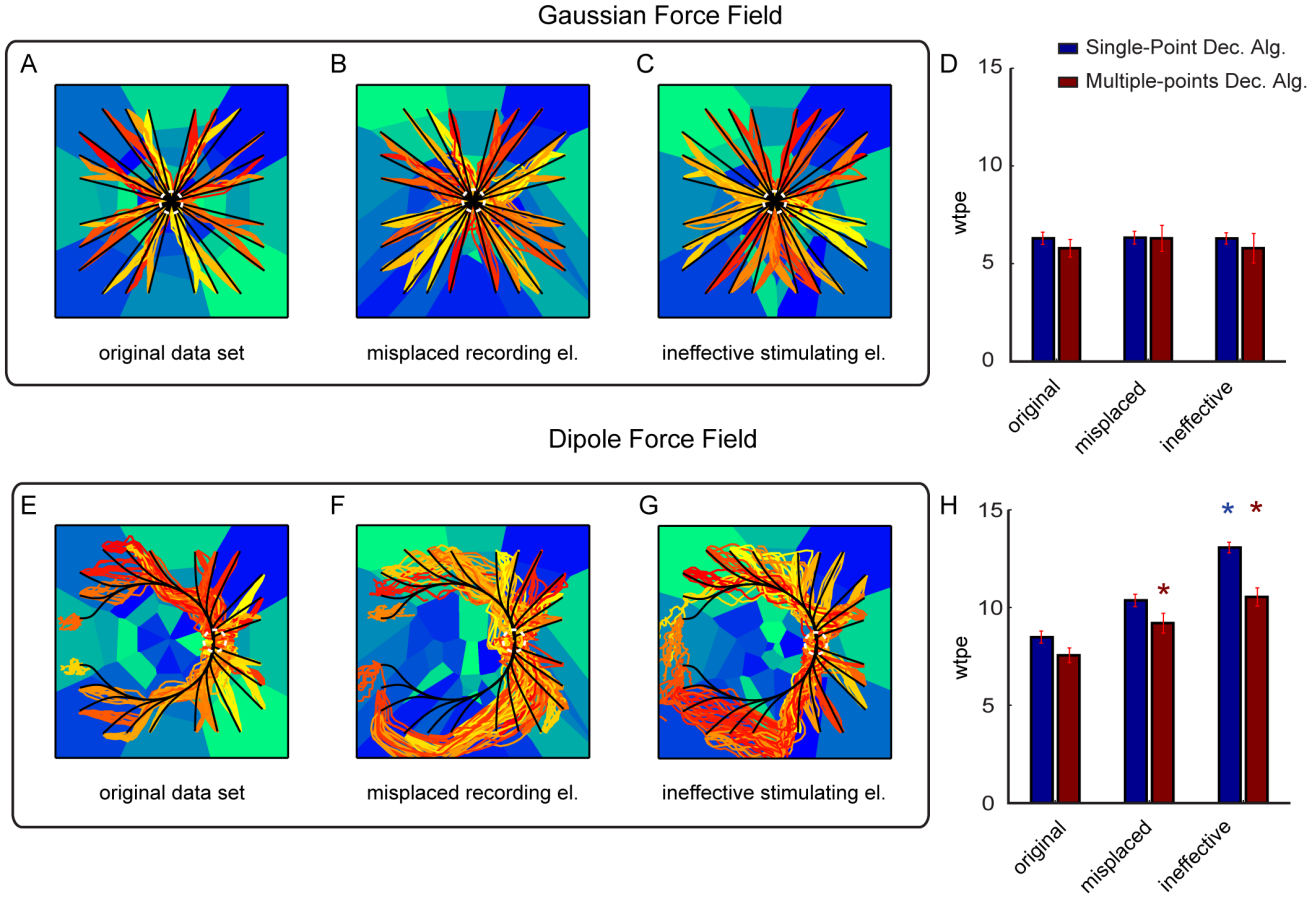
Performances of the ndBMI when simulating one malfunctioning recording and stimulation electrode. The actual trajectories of the Multiple-points algorithm (red-tonality lines) and the ideal trajectories (black lines) superimposed to the sensory regions (blue-tonality areas) by using data set 6 (i.e. 32 stimulating electrodes) and two force fields obtained by utilizing respectively (A, E) the original data set, (B, F) a data set simulating a misplaced recording electrode and (C, G) a data set simulating an ineffective stimulating electrode. (D, H) Bar chart representing the average within-trajectory position error (*wtpe*) of the trajectories obtained simulating a misplaced recording and ineffective stimulating electrode compared with the one simulated by using the original data set. The * denotes that *wpte* depended significantly on the malfunctioning electrodes conditions(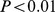 one-way ANOVA) and is placed in correspondence of the malfunctioning conditions for which *wpte* was significantly different from the original data set (Tukey hsd 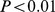).

We then tested two other scenarios, in which we simulated that one of the recording electrodes (Figure 10B,F) or one of the stimulating electrodes (Figure 10C,G) were not effective. In the former scenario, we simulated a misplaced recording electrode that records activity outside the region affected by stimulation (and thus reported a constant number of spikes regardless the stimulus delivered). In the latter scenario, we simulated an ineffective stimulating electrode that failed to elicit any spatially localized modulation of responses in the recording electrodes. The Gaussian force field interface was extremely robust (no significant decrease in performance) in both the simulated scenarios. In contrast, the Dipole force field interface exhibited a decrease in performance (increase in wpte error) in both the simulated scenarios of electrode degradation. For the Dipole Field, the error with a defective recording electrode was 40% larger than the one of the clean simulation (Figure 10H).

**Figure 11:**
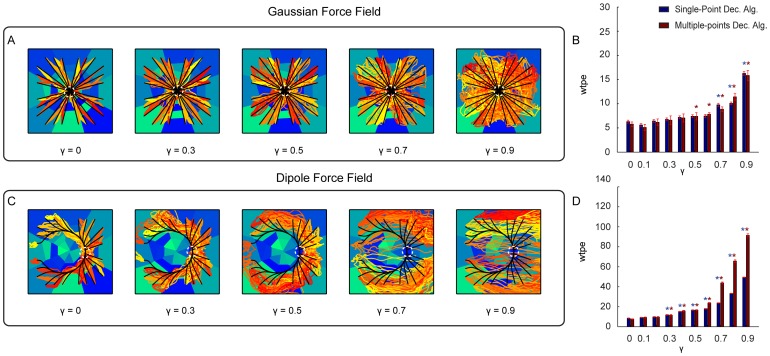
Performance of the ndBMI when reducing information about the stimulation pattern only during the testing phase. (A,C) Ideal (black lines) and actual (red-tonality lines) trajectories of the Multiple-points algorithm superimposed to the sensory regions (blue tonality areas). Uncorrupted data were used during the calibration procedure 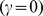 and corrupted data were used during the testing phase (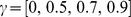), (B,D) Bar chart of the average within-trajectory position error (*wtpe*) between the ideal and actual trajectories calculated for different value of 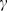. The * denotes that *wpte* depended significantly on 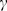 (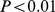 one-way ANOVA) and is placed in correspondence of the values of 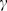 for which *wpte* was significantly different from the “reference” condition with 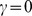 (Tukey hsd 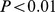).

As previously stated in the above simulations the same degree of degradation was applied to calibration and test data. However, a situation that may be encountered in real experiments is when the recording or stimulation system works well during calibration but then degrades during testing. This may be the case with alterations in signal quality during chronic recordings. We simulated this situation by first calibrating the interface on uncorrupted data (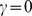) and then testing the interface on corrupted data 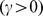. The performance of the interface in this scenario is reported in Figure 11. It is interesting to compare this case of corruption of neural signal only during testing with the previously reported case (see Figure 7) in which the same corruption was present both during calibration and during testing. With respect to this previous case, in the case of corruption during testing only we found a mild decrease of performance of the interface for moderate values of corruption (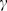 in the range 0.3–0.7), probably because of the mismatch between the neural firing properties between calibration and testing. However, for extreme values of corruption 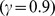 the interface was more robust when degradation happened only during testing. This, in our view, happened because its sensory regions, being computed from non-degraded activity, were of high quality for all values of 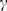 (see Figure 11). For large degradation of neural responses, the beneficial effect of the uncorrupted sensory regions outweighed the negative effect of the mismatch between calibration and testing firing statistics.

### Addition of Volitional Control to Shift at will the Position of the Target Region

In the above simulations, we considered a neural system whose motor cortical response properties depended only upon the specific pattern of electrical stimulation to the sensory region. In this configuration is that the BMI implements, in an essentially automated way, only the particular behavior (for example, reaching a fixed target region chosen at the stage of calibration) implied by the desired force field that was programmed into the sensory and motor interfaces. This raises the question of how the brain may control and modulate at will the operation of the interface, for example by shifting or reaching the target regions at will. We addressed this issue by simulating the presence (during test trials) of a volitional control input aiming to shift the equilibrium position of the point mass from the original equilibrium point of the force field set during calibration to a new location. In fact, if 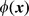 is the force fields programmed by the calibration of the BMI, a simple change may be imposed by a transformation 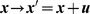 where 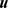 is a component introduced by volitional activity impinging upon the recorded population from structures outside the pathway between stimulation and recording arrays. Accordingly, the force field is changed by volition to 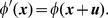 To describe how we can introduce such a volitional modulation, let us consider the case in which, as described above, calibration is made on data that do not contain any volitional component. After the calibration, the sensory interface maps a position 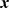 into a pattern of stimulation that evokes neural activity with a spike count output 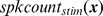. This is the neural response caused by the stimulation array as described in Section *Simulated neural data*. Let us now assume that during test trials the neural activity recorded from motor cortex contains an additional component expressing a volitional command, for example due to inputs to the considered motor region that do not originate from the sensory region stimulated by the interface. For simplicity, we assume that the volitional component is additive. In this case, the spike count obtained after electrical stimulation is made of a stimulus-evoked component 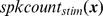 and of a volitional component 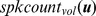 that is independent from the current state of the device and reflects only the intention of the subject to modify the trajectory:
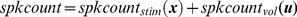
(23)


Consider now for simplicity the case of the Gaussian force field, and suppose that the purpose of the voluntary addition is to move the target equilibrium position to a new point different from the center of the Gaussian force field. The addition of a volitional constant firing rate term to motor cortical activity can be used to shift the position of target region at will. To do so, it is enough to add a term which, if emitted in isolation (without noise and without the firing rate emitted by the non-volitional part), would lead to a force pointing toward the target from the equilibrium point. In the case of the radial field as in the example, this is simply computed as
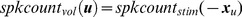
(24)where 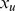 is the volitional target position. In this way, the translation of the total firing rate given by the motor interface tends to provide a constant shift of the dynamics toward the desired equilibrium point.

**Figure 12:**
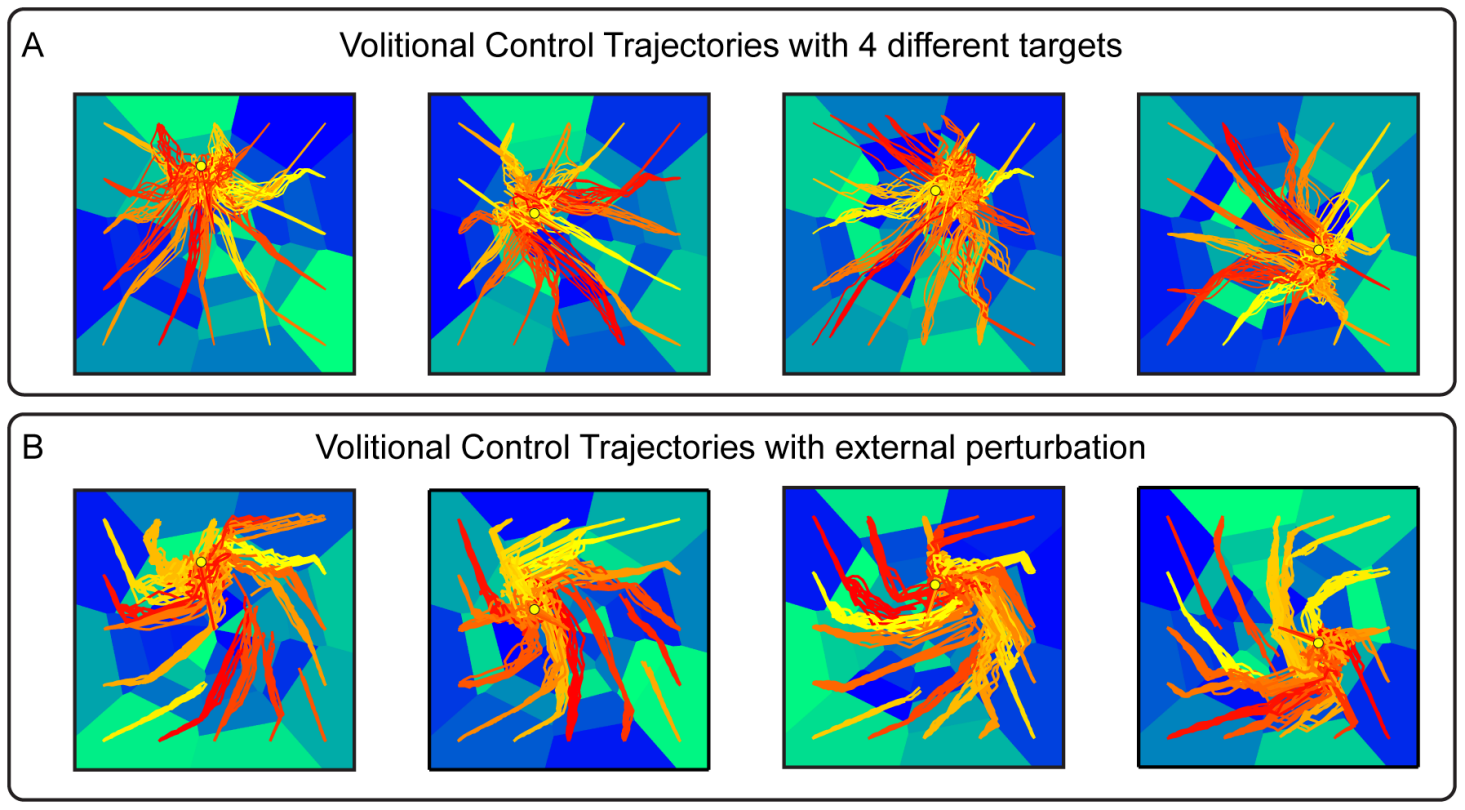
Performances of the ndBMI when adding a voluntary command and an external perturbation. Actual trajectories of the Multiple-points algorithm (red-tonality lines) superimposed to the sensory regions (blue-tonality areas) obtained using data set 6 (32 stimulating electrodes) obtained by adding (A) a voluntary input signal that drives the system toward 4 new equilibrium points (yellow dots) and (B) an external perturbation in the form of skew-symmetric velocity dependent force field.

Figure 12A reports the trajectories that we obtained by adding a volitional firing rate term (computed as described in equation 24 given the chosen position of the new wanted equilibrium point) to an ndBMI with a Gaussian force field. We ran this algorithm using the Multiple-points decoding algorithm. Since the 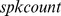 function is defined as constant over sensory regions rather than having a different value for each point of the state space of the actuator, we chose as volitional target positions only positions onto which one or more of the calibration trials were projected. The number of spikes due to the volitional input was generated in each trial using a Poisson distribution with the average spike count specified by 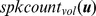, and added to the spikes generated by the intrinsic sensory to motor area mapping described previously. In each of the 4 panels of Figure 12A we report results for 4 different volitional components, which lead each to a different desired equilibrium point (represented by a yellow dot in the domain). In each of these 4 cases we generated a set of trajectories originating from 16 different starting points. These trajectories should be compared with those shown in the middle panel of Figure 6B, which show trajectories with the same interface parameters but without the addition of voluntary firing rate component. In agreement with the intuition explained above, the effect of the voluntary input is a shift of target toward different locations surrounding the center of the workspace.

We further tested the stability of the volitional shift of target by adding an external perturbation to the force field, in the form of a skew-symmetric velocity dependent field:
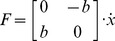
(25)


The effect of this field is to apply a force orthogonal to the movement and proportional to its instantaneous speed. Several studies of motor learning [34], [35] have employed this type of force perturbations for challenging arm movement stability and investigating adaptive control. The simulation results in Figure 12B illustrate the stability of the volitional perturbation, as the motion of the point mass converges toward the attractors encoded by the volitional control despite trajectories are deflected in a general counterclockwise pattern.

## Discussion

### Laying the Foundations for Bidirectional BMIs

The idea of controlling robotic devices by brain activities has been driven by two different perspectives. One is the perspective of providing people with severe form of paralysis with a new “channel to the world”. The other is to provide researchers in Neuroscience with a new family of experimental tools for answering basic questions, such as how the known mechanism of neuroplasticity may provide the physiological foundation for learning and memory. While in the last decade there have been substantial advances on both perspectives, most practical and conceptual challenges remain widely open. Research in brain-computer or brain-machine interfaces is attracting a growing number of scientists from different disciplines and now forms a sizeable scientific community. This community is actively exploring all aspects that affect the information flow of a BMI system, from improvements in the materials that are used to build the electrodes to the development of new decoding algorithms that more reliably transform the neural signals into motor commands for an artificial limb.

In this context, the classical scheme of a brain-machine interface includes neural sensors to record motor signals [36] and decoders to extract the motor intents of the subject from the available neural signals [4], [37]. This approach, although successful [38], still considers any sensory feedback as a separate channel, conceptually divided from the decoding of motor command and necessary only in cases where natural feedback is lacking. Another aspect that is missing in this approach is that by directly translating motor cortical signals into a desired movement trajectory, one ignores the functionality of sub-cortical structures, such as the spinal cord, that also participate in the natural production and control of motor behavior. In recent years these two aspects have been explored in a more systematic way obtaining systems and methodologies in which closing the loop between the brain and the artificial object becomes a fundamental aspect [8], [9], [39]. Also the possibility of using the decoded signal to stimulate the spinal cord directly to generate a movement is a topic that has been addressed [40]. To explore how to bring together these two key research directions, here we developed a proof of concept for a novel approach to brain-machine interfaces. Our approach is based on two main concepts: the interface creates a closed loop system in which brain activities from a motor area are decoded into a force acting upon the external device and the state of the external device is encoded into an electrical stimulus delivered to a sensory area, and the encoding and decoding components of the interface are set-up concurrently, so as to approximate a desired force field of arbitrary form.

The final output of the brain-machine interface is determined by three factors, namely i) the encoding and decoding rules, ii) the external environment, which contributes to establishing the state of the controlled device, and iii) the inputs to the recorded neurons other than those caused by the electrical stimulus. The latter includes any random background activity impinging on the recorded population as well as activities driven by volitional commands. Thus, the resulting behavior is a combined effect of the approximated field, neural noise, environmental influences and voluntary control. In this respect, our approach extends the more conventional BMI methods based only on decoding the output activity as a desired state of the controlled device. In these methods, the voluntary commands modulate the firing of the output neurons under the assumption that these activities are the only determinant of the state of motion of the external device. If a perturbation takes place, it can only be detected by vision (or other sensory stimuli) and corrected based on some high order voluntary process. Instead, in our approach some aspects of the response to external influences are encoded in the desired field and can be modulated by higher order voluntary activity.

### Force Fields as Motor Control Policies to Control Dynamical Systems

In this new framework, our BMI does not translate a motor intention into a particular movement, but rather into a specified “dynamical behavior”. By this term we mean a prescription of how the controlled system, for example an artificial limb or in our simpler implementation a point mass, interacts with the environment. The mechanical interaction between a control system and its environment is governed by the exchange of power, specified by effort and motion variables. The controller/environment interaction takes two canonical forms [41], [42], i.e. a) the environment dictates the state of motion (position and velocity) and the controller responds with a force (i.e. the controller is an impedance and the environment an admittance), or b) the controller sets a state of motion and the environment responds with a force. Considerations on the passive mechanics of the musculoskeletal system suggest that the first case is a more plausible description of biological control policies [43]. In fact, earlier experimental studies on spinalized frogs [15], [44], [45] and rats [46] demonstrated that activation of spinal cord interneurons results in a field of forces that drive the limb along different trajectories, depending on the state of motion in which the limb is placed by the interaction with the external environment. This is a natural description of motor control, if one considers that muscles are spring-like elements establishing (non-invertible) mappings from a limb’s state of motion to a resulting viscoelastic force. Since forces are inherently additive, the combined actions of multiple muscles result in the summation of their force fields. Therefore, the description of motor control policies as force fields has the important property of providing a compositional mechanism for the generation of a large repertoire of behaviors from the sum of a relatively small number of force-field “primitives” [20].

We have developed the computational analysis in parallel with ongoing electrophysiological experiments [21]. For this reason, we considered brain regions that are known a) to play functional role in receiving sensory information and dispatching motor commands and b) to have a pattern of interconnections. Furthermore, cortical regions are significantly easier to access and manipulate experimentally than the spinal cord or other subcortical regions. We focus on the transformation that is carried out by the neural circuits intervening between stimulation and recording arrays, because we expect that the output of the recording array can also be modulated by volitional influences. In a recent study [21] on anesthetized rats we tested the first prototype of a bidirectional BMI in which motor cortical activities were linearly mapped to force vectors acting upon a simulated point mass and the position of the point mass was encoded into an electrical stimulus delivered to the somatosensory cortex. With this approach, we were able to approximate a linear force field converging to a single equilibrium location. However, a limiting constraint for that approach was the requirement that the relation from position to force was invertible. This was due to the fact that the approximation procedure first defined the domain upon which the applicable forces were defined and then the forces produced by each stimulation pattern were mapped during the calibration to the locations at which these force were to be applied. This approach drastically limited the repertoire of feasible control policies, a limit that is now removed by the current algorithm. Here, the position of the controlled object maps to an electrical stimulus and the response to this stimulus - which is also open to other inputs, such as volitional activities - is decoded in the location at which the force is calculated based on the predefined force field. Therefore, the information flow from position to force never needs to be inverted. The possibility to represent a broader repertoire of fields is of essential importance since relevant motor behaviors and control policies are not limited to force fields converging toward a single equilibrium point. More generally, force fields also offer an adequate description of the stretch reflex, first described by Sherrington [47] and of spinal pattern generators. The latter induce a cyclical motion of the limbs. Grillner and coworkers [16] offered a compelling model of locomotion pattern in the lamprey, and in both cases the rhythmic activity is sustained by a phase-shift between the state of motion and the consequent forces.

In the current implementations we have only considered a fixed force field, established by coordinating the calibration of the sensory and motor interfaces. This approach however is open to future developments in which, for example, multiple force fields, with different structures, are implemented in parallel. In this case, the voluntary activity could effectively generate a larger repertoire of behaviors by establishing weighted combinations of these force fields.

### Limitations of the Simulated Neural Systems used to Test the ndBMI

The performance and robustness of our new ndBMI was tested and validated with a simple descriptive model of stimulation and neural responses in the sensory-motor loop inspired by the cortical sensory motor loop in the rat whisker system. The model captures, in an idealized and simplified way, two observed features of cortical responses: Poisson variability of spike train responses and topographic organization between stimulated and evoked activity.

Poisson processes are simplified model of neural responses, because they neglect any form of auto- and cross-correlation among the spike trains. Yet this model is a relatively realistic one, in that it correctly represents the approximate order of magnitude of the trial-to-trial variance of cortical responses, which (like in the Poisson model) is relatively close to its mean. An important question for practical applications of this technique is whether the simplified nature of the Poisson model may limit the inferences that we can make about the performance of the algorithm on real data. The first consideration is that the algorithms seem to be remarkably robust; we found they were robust even to very large degradations of the information carried by neural recordings. In many respects, the assumptions of the Poisson model are conservative with respect to the information content of neural activity, as cortical responses often report sub-Poisson variance [32], [48], including in the animal models that we use for developing bidirectional BMIs [21]. Moreover, autocorrelations and cross-correlations of neural activity found in real data usually have small effects on the information content, compared to what would be expected if statistics followed the Poisson model [49]–[52]. Whatever the exact response statistics of the neurons under consideration, it is important to bear in mind that the ndBMI algorithms presented here do not rely at all on the assumption that the data are distributed according to a Poisson distribution. They can operate with correlated data and can capture information or metric structure encoded by correlated firing, if present.

An organization of motor neural responses that preserves the geometry of sensory stimulation patterns is also supported by data in the sensory motor whisker system [29]. While the precision of topography implemented in our simulations may be difficult to obtain in experimental situations, we demonstrated a strong robustness of the algorithm to loss of specificity of responses, and the algorithms do not assume or rely on topography of responses at all.

Therefore, although of course a full validation of the capabilities and potentials of the algorithms presented here will require their future testing on real data, our expectation is that the simulated study presented has a sufficient realism to investigate and validate the computational properties of our algorithms.

### Considerations on Volitional Control

In the prevalent BMI system models [4], [6], [37], [38] neural activities are decoded by some algorithm, which dictates the instantaneous position of a controlled device, such as a robotic arm or a cursor on a computer monitor. Here, we suggest that the brain activities, instead of specifying a position, contribute to determine the force vector that is applied to the device. We stress the word “contribute” instead of “dictate” because the net force generated by the interface results from the combination of the volitional command with the state-dependent component from the sensory interface. In this study we have limited the analysis to demonstrate through simulation that it is possible - in principle - for the volitional activity to shape the force field so as to drive the device to a desired target. In a physical implementation, this possibility rests upon the ability of the brain to address by volitional activity the neural population that is being recorded and to learn how to shape the field according to the behavioral goal. This is of course a major open challenge, whose possibility of success is supported by the demonstrated ability of the brain to shape by volition the activities of specific target neurons [53], [54].

Our approach is based upon generating a field by closing the loop between recording and stimulation. With this, we do not deny the validity of having control system dynamics external to the brain. On the contrary, while here we consider rather primitive forms of external dynamics (a point mass in viscous fluid) more advanced applications can be considered, including some external controller. The issue of using neural or artificial controllers is similar to the debate on whether using M1 activities to control the dynamics of a robotic arm, instant by instant, or decode higher level commands from the posterior parietal cortex and have a robot implementing the desired goals. We feel that at this time it is important to explore all these alternatives. The rationale for our approach over one based on an external device is that we want to consider the possibility for the brain to access within its volitional centers, the same sensory information that, through the dBMI, causes the activation of the recorded neurons.

The bidirectional interface proposed here can generate patterns of automatic sensory-motor responses in a way analogous to spinal [55] and supraspinal [56] reflex mechanisms. With this approach, we are implicitly assuming that the alert brain would be able to modulate the output of the interface, thus adding a volitional decision-making component to the picture. This assumption does not rest on blind faith, but on a repertoire of examples in which the brain learned to focally control the output of individual neurons and neuronal populations for the operation of external device [3], [4], [37]. In these previous examples, brain activities were directly decoded into some state variable, like the desired position of a manipulator or of a cursor. Here, instead, we propose that the volitional neural activities modulate the field established by the bidirectional interface. We demonstrated in one example that this may lead to effective control over the desired position of the target. However, this volitional signal combined with the field encoded by the interface does not only establish a target position but effectively a whole family of trajectories. Furthermore, the field properties offer a mechanism to ensure stability of the resulting motions, which can reach the desired target in spite of external perturbing forces acting on the controlled device. The repertoire of possible behaviors is not limited to movements directed toward an equilibrium position. We have shown that the inherently additive nature of force fields allows the programming of combinations of multiple goals, such as reaching a target while avoiding an obstacle. Furthermore, the structure of the force field can be shaped to drive the controlled device in cyclical and other behaviors. Ultimately, what we are proposing in this example is to move one step forward toward the implementation of bio-mimetic control mechanisms, by reproducing a structure of control processes that is analogous to the interaction between brain and spinal cord in vertebrates. This interaction is based on the seamless integration of voluntary commands with a peripheral neuromechanical structure. As a result our movements are endowed with adaptive properties that emerge from the coupling of neural information processing with the physical properties of the environment and of the musculoskeletal apparatus.

In some ways, our approach is an attempt at effectively emulating the spinal cord in the portion of the brain that connects the recording and stimulating electrodes. While we have already obtained some experimental validation of this approach [21], we do not suggest that we are ready for clinical application. At this time, we are merely proposing to coordinate the input and output interfaces based on a shared goal, the generation of a force field to be modulated by central activities. Would such a system eventually disrupt the normal operation of the brain? Indeed, it would not be wise to take over brain regions that are normally functional. The brain machine interface has been proposed as a paradigm for severely paralyzed stroke and spinal cord injury survivors. Looking at a future application of our system, as of any other BMI, it will be critically important to avoid interference with functional structures of the sensory-motor apparatus.
